# From Bench to Bedside and Beyond: Therapeutic Scenario in Acute Myeloid Leukemia

**DOI:** 10.3390/cancers12020357

**Published:** 2020-02-04

**Authors:** Carmelo Gurnari, Maria Teresa Voso, Jaroslaw P. Maciejewski, Valeria Visconte

**Affiliations:** 1 Department of Translational Hematology and Oncology Research, Taussig Cancer Institute, Cleveland Clinic, Cleveland, OH 44195, USA; carmelogurnari31@gmail.com (C.G.); maciejj@ccf.org (J.P.M.); 2 Department of Biomedicine and Prevention, University of Rome Tor Vergata, 00133 Rome, Italy; Voso@med.uniroma2.it; 3 Neuro-Oncohematology Unit, Fondazione Santa Lucia, Istituto di Ricovero e Cura a Carattere Scientifico (I.R.C.C.S.), 00143 Rome, Italy

**Keywords:** acute myeloid leukemia, FLT3, IDH1, BCL-2, Hedgehog pathway

## Abstract

Acute myeloid leukemia (AML) is a heterogeneous group of clonal disorders characterized by abnormal proliferation of undifferentiated myeloid progenitors, impaired hematopoiesis, and variable response to therapy. To date, only about 30% of adult patients with AML become long-term survivors and relapse and/or disease refractoriness are the major cause of treatment failure. Thus, this is an urgent unmet clinical need and new drugs are envisaged in order to ameliorate disease survival outcomes. Here, we review the latest therapeutic approaches (investigational and approved agents) for AML treatment. A specific focus will be given to molecularly targeted therapies for AML as a representation of possible agents for precision medicine. We will discuss experimental and preclinical data for FLT3, IDH1, BCL-2, Hedgehog pathway inhibitors, and epitherapy.

## 1. Introduction

Despite the progress in treatment and supportive therapies, the outcome of patients with adult acute myeloid leukemia (AML) remains dismal with only about 30% of patients becoming long-term survivors [1,2].

Globally, AML accounts for the highest percentage of leukemic deaths and every year 4–5 per 100,000 persons are expected to develop this disease with a peak of incidence over the sixth decade of life and a slight male predominance [3]. During the last few decades, a mild improvement has been registered in terms of overall survival (OS) for patients with AML, although no substantial changes have been made in the backbone of induction therapies, still relying on the well-known chemotherapy “3+7”regimen (7-day continuous infusion of cytarabine and daunorubicin at various dosages on days 1 to 3) first described in 1973 [4]. Later on, every effort made to overcome AML resistance has been a mozartian “variation on theme” of studies investigating different dosage and posology of the anthracycline-based regimen. Therefore, the success in OS rates is probably explained as the result of a better management of the antimicrobial stewardship and hematopoietic stem cell transplantation (HSCT) techniques, as well as the deeper knowledge of minimal residual disease detection [5,6,7,8]. The recent progress in understanding AML pathogenesis and the identification of new candidate driver mutations revealed considerable genomic heterogeneity and complexity of the disease and defined new diagnostic and prognostic markers, paving the way for the discovery of new potential targeted therapies [7]. However, as the population is living longer, two more concerns are emerging as new unmet clinical needs in AML epidemiology: (1) Higher numbers of older patients, harboring unfavorable cytogenetic features probably reflecting antecedent myeloid disorders which underwent clonal evolution; (2) higher numbers of therapy-related AML in long-term cancer survivors [9]. Both these clinical scenarios are characterized by poor prognosis and refractoriness to conventional chemotherapy. In this light, the identification of new actionable targets for the therapy of these diseases has been considered as a high priority.

In the last 10 years, the U.S. Food and Drug Administration (FDA) approved several new pharmacologic agents for the treatment of AML, enhancing the plethora of treatments in the new millennium scenario. A full description of the numerous drugs and clinical trials of the recent years is outside the scope of this manuscript; however, we highlighted the crucial role of potential direct and/or indirect therapeutic targets (gene mutations, deregulated pathways) for AML treatment.

## 2. Fms-Related Tyrosine Kinase 3 (FLT3) Inhibitors

Fms-related tyrosine kinase 3 (FLT3) is a cytokine receptor regulating cellular differentiation and proliferation through multiple activation pathways (e.g., PI3K/AKT: Phosphatidylinositol-4,5-bisphosphate 3-kinase/v-akt murine thymoma viral oncogene homologue; JAK/STAT: Janus kinase/ signal transducer and activator of transcription; MAP kinases: Mitogen-activated protein) [10]. In 1996 Nakao et al. reported for the first time an internal tandem duplication in the juxtamenbrane domain (FLT3-ITD) gene, located on chromosome 13q12, accounting for about 30% of cases of adult AML [11]. FLT3-ITD-mutated AML is most frequently characterized by normal karyotype, myeloblastic/monoblastic differentiation, poor prognosis with a high leukemic burden at diagnosis, and shorter OS, if compared with FLT3-ITD-unmutated patients, due to its strong relapse tendency [12,13]. The mutant-to-wild-type allelic ratio and the presence of concomitant nucleophosmin (NPM1) mutations influence the prognosis of this subset of patients [7] defining different subclasses of FLT3 and/or NPM1-mutated leukemias. Conversely, the role of tyrosine kinase domain (TKD) point mutation D835 is still controversial and not entirely understood. However, mutational frequencies account for 25% of all FLT3-mutated leukemias and about 7% of all adult AML [14]. The recent ELN 2017 classification opened the road for the clinical management of this setting of patients according to the FLT3-ITD allelic ratio. The definition of allele ratio as the ratio of the areas under the curve (peak height) of FLT3-ITD compared to the FLT3 wild-type product became a criterion of assignment of the risk of relapse and helped in guiding post-remission treatment decision. Indeed, allele ratio less than 0.5 is an indicator of a more favorable prognosis. Of note, this classification did not take into account the use of FLT3 inhibitors used during induction treatment, making important future clinical trials. Moreover, the decision between consolidation chemotherapy and HSCT as post remission strategies after FLT3 inhibitors, is a current matter of concern given the fact that these compounds possibly delay relapse without affecting OS, thus maintaining the obtained remission (e.g., sorafenib in the context of post HSCT maintenance) [7,15].

Two generations of FLT3 inhibitors have been developed and explored so far in preclinical and clinical settings (Table 1). The first generation includes a broad-spectrum tyrosine kinase inhibitor (TKI) with a cascade activity involving the proto-oncogene receptor tyrosine kinase c-KIT, the platelet derived growth factors-PDGF, and the vascular endothelial growth factor-VEGF rather than influencing FLT3 alone. This characteristic has led the use of these compounds in several other non-hematological cancers (e.g., gastrointestinal stromal tumors) [16]. The second generation has been developed in order to more selectively target FLT3, possibly avoiding “off target” effects. Another general classification based on the conformation of the targeted kinase can divide FLT3 inhibitors into type I by blocking both the active and inactive conformation of the receptor (binding to the gatekeeper domain of FLT3 near the ATP-binding pocket or the activation loop) and type II by binding to the hydrophobic region of the ATP-binding domain only when the receptor is in its inactive conformation [17]. This latter classification is particularly important because it clarifies the reason why FLT3-mutated AML resistant clones are more prone to arise after type II inhibitors treatment and to acquire TKD point mutations due to the more narrow and specific selective pressure of these compounds [18].

Midostaurin is the first FDA approved FLT3 inhibitor. This first-generation compound has a broad kinase activity profile and was originally developed for solid tumors. Midostaurin has a potent antiblast activity and is now part of the treatment of FLT3-mutated patients during 3+7 induction scheme and post remission regimens. The Randomized AML Trial in FLT3 Young patients (RATIFY) is a multicenter, randomized, double-blinded, and placebo-controlled phase 3 pivotal trial enrolling 717 patients with de novo AML and age less than 60 years. The study compared midostaurin 50 mg BID to placebo in combination with the standard induction regimen 3+7, followed by consolidation and maintenance in patients harboring both FLT3-ITD and TKD mutations. This combination resulted in a better OS and event free survival (EFS) for the group receiving the drug, yielding to FDA approval [14]. However, FDA has not licensed midostaurin for maintenance therapy after HSCT and clinical trials are still ongoing evaluating the role of this agent after HSCT.

Stone et al. [14] administered daunorubicin at a dosage of 60 mg/m^2^. It is a current practice to use anthracycline ranging from 45 to 90 mg/m^2^, according to the fitness of patient, mostly dependent on the age and many studies have been conducted so far to understand the best dosage. However, it has to be noted that midostaurin and daunorubicin share common toxicities (e.g., GI) suggesting more precautions and low anthracycline dose if used in combination [20]. Moreover, midostaurin is bypassed by CYP3A4 (cytochrome P450 3A4), the same cytochrome involved in the metabolism of antimold agents, commonly used as prophylaxis during induction in this group of patients. Therefore, it is suggested that this particular pharmacological interaction is considered when choosing the best induction treatment and supportive care for patients with AML, individualizing the medical choices and avoiding the paradigm of “one size fits all” [21].

The second and more selective generation of FLT3 inhibitors includes quizartinib, crenolanib, and gilteritinib. Quizartinib (AC220) is a potent FLT3 inhibitor with a weak non FLT3-ITD TKI activity. Thus, TKD mutant clones represent a well-known escaping mechanism after quizartinib treatment [22]. Quizartinib was first studied in the relapsed/refractory (R/R) setting as a single agent (QUANTUM-R trial) and is currently tested in a multinational, double-blinded, randomized, placebo-controlled study in combination with standard induction therapy for newly diagnosed adult patients with AML who will receive it for up to one year of maintenance (also after HSCT, NCT02668653). Moreover, other studies are still ongoing evaluating the combination of quizartinib and hypomethylating agents (HMA) or low-dose Ara-c (LDAC), in the setting of patients classified as not-fit for intensive chemotherapy [23]. A significant class effect, known at different degrees in the case of every FLT3 inhibitor, is the QTc prolongation that has been addressed by using an appropriate dosage of 60 mg once daily as derived by the QUANTUM-R study [24]. 

Similar to quizartinib, crenolanib is another potent and selective FLT3 inhibitor, although it retains activity against TKD mutations, overcoming the known mechanism of resistance of the previous second generation drug. Resistance to crenolanib is acquired in the case of F691L gatekeeper mutations, targeted by other TKI inhibitors such as ponatinib or new compounds still under investigation such as PLX3397 and cabozantinib [25,26]. Crenolanib has been studied alone in the R/R AML setting with an overall response rate (ORR) of 50% and is now on study in combination with standard induction chemotherapy also compared to other FLT3 inhibitors such as midostaurin [27,28].

In 2018, the FDA approved gilteritinib for the treatment of R/R FLT3-mutated AML after the promising results of the ADMIRAL trial, a randomized, open label, multicenter phase III study comparing gilteritinib or conventional chemotherapy for R/R FLT3-mutated AML patients [29]. This new agent has a potent activity against both ITD and TKD mutations, blocking FLT3 and also AXL activity, whose increased expression has been attributed to a mechanism of chemo-refractoriness [30]. As other FLT3 inhibitors, gilteritinib has been tested alone or in combination with HMA with promising results. Many studies are currently ongoing by combining this drug with standard chemotherapy regimens or with new agents (NCT02752035, NCT02236013).

Despite the development of all these new compounds, there is still room to improve survival outcomes in FLT3-mutated AML. Although FLT3 inhibitors are known to provide a viable blast reduction and maintain remission state, many patients are resistant or relapsing after treatment due to various possible mechanisms.

The first remarkable result is that FLT3 inhibitors are very useful for reducing the peripheral disease burden although this does not necessarily translate into a similar effect on bone marrow (BM) disease burden. Many studies have proved how the BM microenvironment plays an important role in mediating resistance to FLT3 inhibitors due to the expression of CYP3A4 by stromal cells, providing protection against these new selective drugs by affecting their pharmacokinetics [31]. Moreover, BM stromal cells produce numerous cytokines including CXCL12/CXCR4 (C-X-C motif chemokine 12/C-X-C chemokine receptor type 4) signaling, RAS (Rat sarcoma) kinases, and PI3K/AKT/ mTOR (mammalian target of rapamycin) that seem to play a major role providing the activation of survival pathways that bypass FLT3 receptor [32]. For instance, McMahon et al. demonstrated the emergence of resistant clones acquiring RAS/MAPK pathway mutations in FLT3 positive AML after treatment with gilteritinib [33]. Due to these mechanisms, the use of RAS inhibitors, as well as mTOR inhibitors in this setting of R/R AML is becoming feasible [34]. 

**Figure 1:**
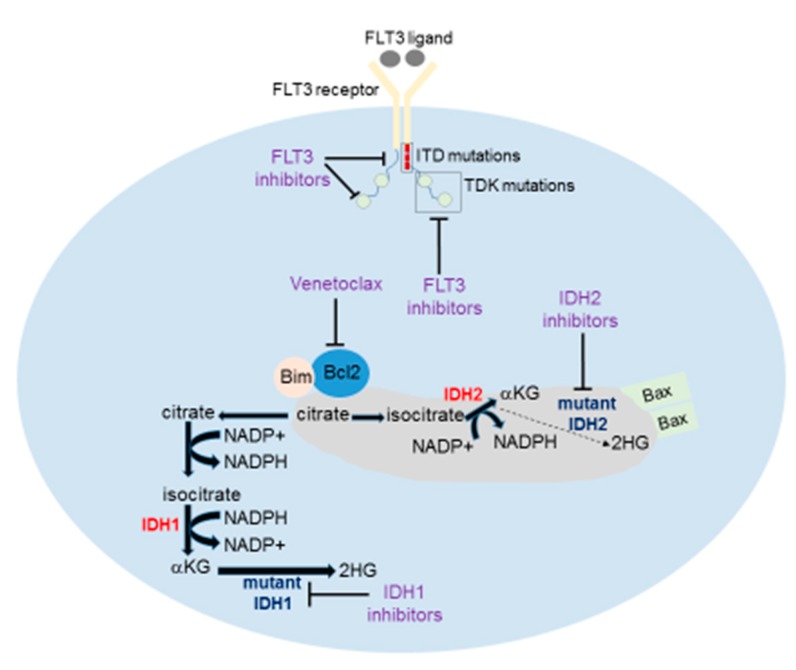
Inhibitors have been designed to target major pathways deregulated in acute myeloid leukemia (Bcl-2), mutant form of metabolic regulatory enzymes (IDH1/2), and proliferation (FLT3). Abbreviations: 2-HG: 2-hydroxyglutarate; α-KG: Alpha-ketoglutarate; BAX: Bcl-2-like protein 4; Bim: Bcl-2-like protein 11; Bcl-2: B-cell lymphoma 2; FLT3: Fms-related tyrosine kinase 3; IDH: Isocitrate dehydrogenase; ITD: Internal tandem duplication; NADP(H): Nicotinamide adenine dinucleotide phosphate; TKD: Tyrosine kinase domain.

Furthermore, FLT3 constitutive active signaling converges to downstream activation of STAT5, also associated with concomitant phosphorylation of FOXO3A (retained into the nucleus) and AXL upregulation, a tyrosine kinase receptor which is higher expressed under quizartinib treatment and hypoxia conditions of the hematopoietic niches [35]. STAT5 activation generates a positive anti-apoptotic signaling through Pim-1 and upregulation of Bcl-2 and its family member MCL-1(myeloid cell leukemia-1) that immortalize FLT3-mutant blasts [36,37] (Figure 1).

Therefore, targeting these pathways with Bcl-2 inhibitors such as venetoclax may represent a way to overcome FLT3-mutated R/R AML. Recent studies have also shown the important role of FOXM1 in R/R AML and particularly in FLT3-ITD-mutated cases where the expression of Forkhead box (FOX) proteins correlates with OS. In vitro studies have shown that inhibition of FLT3-ITD by quizartinib/AC220 also downregulates FOXM1 expression in FLT3-mutated cells suggesting that FOXM1 may be a potential prognostic marker and therapeutic target in AML. Ultimately, secondary mutations in the FLT3 gene often involving single amino acid substitutions have been postulated as a putative mechanism of resistance. These mutations are known to occur specifically in relapsing patients after type II inhibitors treatment and can be targeted by broader TKI such as ponatinib or sorafenib [38]. Most likely these mutations are present at diagnosis in a subclonal configuration (as the case of TKD) and arise after exposure to the selective pressure of a narrow spectrum of FLT3 inhibitors [22].

## 3. Isocitrate Dehydrogenases (IDHs) Inhibitors

Isocitrate dehydrogenases (IDHs) are metabolic regulatory enzymes, recognized as mutated in various types of cancer (gliomas, colorectal and prostate cancer, and hematological malignancies as AML and myelodysplastic syndromes) [39,40,41,42].

In particular, isocitrate dehydrogenases 1 (IDH1) is the cytoplasmatic isoform of a class of dehydrogenases involved in the Krebs cycle whose role is converting isocitrate, generated in the tricarboxylic acid cycle (TCA), to alpha-ketoglutarate (α-KG) [43]. The mutant IDH1 enzyme causes the production of the putative oncometabolite D-2-hydroxyglutarate (2-HG) instead of α-KG, interfering with mitochondrial function. 2-HG is structurally similar to α-KG and inhibits α-KG-dependent enzymes, including members of the ten-eleven translocations (TET) family and the jumonji-domain-containing group of histone lysine demethylases. As a result, 2-HG imbalances the cellular redox potential (lacking of adequate levels of Nicotinamide adenine dinucleotide phosphate-H/NADP-H) and increases histone and DNA methylation, resulting in epigenetic imbalance [43,44].

Marcucci et al. [45] reported hot spots mutations for IDH1 (R132) and IDH2 (R140 and R172), occurring in approximately 8% and 12% of patients with cytogenetically normal AML. The mutations occur in the region of isocitrate binding. Moreover, it has been reported that IDH are frequently mutated in elderly individuals with clonal hematopoiesis of indeterminate potential (CHIP), a condition associated with an increased risk of developing hematologic neoplasms [46]. In AML, their prognostic impact is strictly dependent on the presence of other gene mutations, being the majority of IDH-mutated AML also NPM1-mutated, FLT3 wild-type, and typically present in the elderly.

Recently FDA approved ivosidenib (AG-120), a first-in-class IDH1 inhibitor [47] and enasidenib (AG-221) for IDH2-mutated AML [48]. The former is indicated for R/R IDH1-mutated AML patients or upfront in older patients (75 or older) or with contra-indications for intensive chemotherapy. These results have been provided by the study of DiNardo et al. in a cohort of 179 patients with R/R AML harboring *IDH1* mutation treated with 500 mg oral ivosidenib resulting in an overall response rate (ORR) of 41%, 21% complete response (CR), and 30% hematological improvement [49]. Enasidenib also received approval for adults with R/R IDH2-mutated AML after the promising results of Stein et al. who showed an ORR of 40% and CR rate of 19% in a cohort of 199 patients treated with 100 mg orally daily [50]. This drug is now under investigation in a phase II, multicenter, open label, 2-arm clinical trial (NCT03383575) for untreated MDS/oligoblastic AML (arm A, receiving both enasidenib and azacitidine) and R/R higher risk MDS after HMA failure (arm B, receiving only enasidenib at the standard dose of 100 mg orally). Both IDH inhibitors have a similar spectrum of mechanism of action and side effects. They act as differentiation-promoting rather than cytotoxic agents, being able to differentiate leukemic blasts in maturating cells, causing in some patients a differentiation syndrome (DS) with a clinical picture resembling to that caused by ATRA (all-trans-retinoic acid) in acute promyelocytic leukemia (APL) [51]. Similar to HMA, IDH inhibitors may require several treatment cycles to induce a response suggesting the importance of continuing therapy for at least six months or until progression/intolerable toxicity. IDH inhibitors-related DS occurs in about 15% of patients and includes a clinical scenario of signs and symptoms resembling Montesino’s criteria of APL DS (acute respiratory distress with pulmonary infiltrates and pleural effusion, renal impairment, fever, and peripheral edema with weight gain), occurring at a median time of 30 days after initiation of treatment. The indications for DS treatment are similar to those provided for APL and include dexamethasone, diuretics, and hydroxyurea to manage the leukocytosis caused by differentiating blast cells [52].

Despite IDH inhibitors development, multiple resistances have been described with poorly elucidated mechanisms. As higher 2-HG levels are required for blasts’ proliferation, one peculiar mechanism to maintain its level under IDH selective isoform blocking, is the “isoform switching” from IDH1 to IDH2 and vice versa [53]. Moreover, it has been shown that somatic point mutations of the wild-type allele may occur at different sites and drive refractoriness of IDH-mutated AML [54]. Therefore, future strategies aiming at restoring IDH activity and regulating 2-HG production will be needed. It has been also proved that 2-HG imbalances the epigenetic cellular landscape through inhibition of the activity of cytochrome c oxidase in the mitochondrial electron transport chain, lowering the mitochondrial threshold to trigger apoptosis through Bcl-2 inhibition and, thus, providing a rationale for the usage of Bcl-2 inhibitors [55]. These combinations may overcome the selective pressure and the subsequent resistance provoked by a single agent treatment.

Furthermore, other IDH1 inhibitors, such as olutasidenib, an oral highly potent and selective inhibitor of IDH1 and vorasidenib (AG-881) a pan IDH inhibitor, are now under investigation [56]. In particular, olutasidenib (FT-2102) has been tested as a single agent and in combination with HMA in a phase I/II trial in R/R AML and in naive patients ineligible for standard therapy with OR of about 40% in both patients subtypes. This drug has been able to induce a mutation clearance (VAF < 1%) in a subset of patients and a rapid reduction of 2-HG by the end of cycle 1 (NCT02719574) [57]. 

Lastly, other nontargeted strategies for IDH1 mutant AML may include PARP inhibitors such as olaparib and talazoparib, already tested in IDH positive gliomas and in studies using AML cellular models [58]. Since glutamine is the main cellular source of α-KG, preclinical and clinical studies are ongoing for testing a new compound named CB-839, an oral glutaminase inhibitor alone or in combination with azacitidine (NCT02071927) (Figure 1).

## 4. B-cell Lymphoma 2 (BCL-2) Pathway Inhibitors

B-cell lymphoma 2 (BCL-2), a member of the BCL-2 family of genes and an integral part of the intrinsic mitochondrial apoptotic pathway, was first discovered in follicular lymphoma harboring t (14;18) abnormality [59]. This pathway is triggered by different cellular stimuli through BH3 (bcl-2 homology 3 protein) that activates BAX (bcl-2-like protein 4) and BAK (bcl-2 homologous antagonist/killer), overcoming BCL-2 anti-apoptotic potential and causing a permeabilization of the outer mitochondrial membrane. The subsequent release of cytochrome c and SMAC (second mitochondria-derived activator of caspases) allows an interplay between caspases 9, 3, and 7, leading to apoptosis, via SMAC-mediated XIAP (X-linked inhibitor of apoptosis protein) blockade [60]. Thus BCL-2 is a pivotal negative regulator of apoptosis, playing an important role in AML transformation, survival, and resistance. 

BCL-2 expression has been found deregulated in AML [61]. Particularly it was higher in M0/M1 FAB subtypes while it was underexpressed in M5, without showing effects on prognosis in terms of OS and leukemia-free survival (LFS). A possible explanation is that BCL-2 overexpression might be due to the undifferentiated phenotype of M0/M1 AML blasts specifically being upregulated in leukemic stem cells (LSCs) [62]. These “rare and quiescent” LSCs are particularly resistant to conventional AML therapy and their BCL-2 overexpression may be selective targeted. 

Since the development of the antisense nucleotide oblimersen (G3139) in 2003, many efforts have been made to discover new drugs targeting BCL-2 pathway. However, many compounds have been tested in AML preclinical and clinical setting without promising results until the discovery of ABT-199 (venetoclax) [63]. The previous drugs were characterized by a lower selectivity for BCL-2, e.g., involving BCL-X_L_ (B-cell lymphoma-extra large) with consequent side effects such as thrombocytopenia [64]. On the contrary, venetoclax is a highly selective and potent oral BCL-2 inhibitor approved in 2016 for the treatment of chronic lymphocyte leukemia (CLL) with 17p deletion [65]. 

Preclinical data suggested that this new drug targeting BCL-2/tp53 apoptotic pathway may be useful also for AML as a single agent or in combination with other backbone therapies [66]. Konopleva et al. first used venetoclax as a single agent in R/R AML in a phase II study assessing safety and efficacy of this drug with an ORR of 19% [67]. However, Ho et al. showed how heterogeneous AML LSCs are at diagnosis and relapse, paving the way for the rationale of using a LSC-active drug such as venetoclax in the upfront setting [68]. A significant study which led to the approval of venetoclax from the FDA was a phase 1b dose escalation and expansion trial conducted in patients with AML ineligible for intensive chemotherapy. In this study, venetoclax was combined with HMA (either azacitidine or decitabine) in treatment-naïve elderly patients [69]. In total, 145 patients with AML (65 or older) were treated with venetoclax at a dose of 400 or 800 mg (expansion study) with conventional azacitidine (75 mg/m^2^, days 1–7 subcutaneuosly) or decitabine (20 mg/m^2^, days 1–5, intravenously) schedules. Half of the patients harbored poor-risk cytogenetics and median age was 74 years. The ORR was 68% with a CR rate of 73% in the venetoclax 400 mg cohort and was of 67% in the whole cohort. The treatment was well-tolerated and common adverse events were mostly gastrointestinal and hematological and in particular neutropenia, probably responsible for early mortality within 30 days after initiation (despite the paucity of events). This latter event has been in part counteracted by the quick median time to best response of 1.8 months if compared with the average four months with HMA-treated historical controls [70]. Moreover, the subgroups analysis showed that venetoclax is also effective in *FLT3*-positive patients and in secondary AML, as well as in *TP53*-mutated cases (despite the lower incidence—47%—and median duration—5.6 months—of CR). Conversely, *IDH1/2*-mutated patients were particularly sensitive to venetoclax and reached a median survival of 24.4 months. However, venetoclax shares some downsides with other AML targeted therapies; it is metabolized by CYP3A4 hepatic cytochrome alerting the use of concomitant drugs utilized in fungal prophylaxis. Nevertheless, the incidence of fungal infections in this trial was similar to other previous reported studies demonstrating the need of reaching adequate neutrophil counts in a short time as it occurs with this combination therapy. 

Venetoclax is now on study with conventional chemotherapy (NCT03709758), as well with other multi-targeted combinations (e.g., *FLT3* inhibitors or glasdegib- NCT03625505 and NCT03735875) to overcome AML resistance and apply a better personalized pharmacologic approach [71]. Moreover, low-dose cytarabine (LDAC) in combination with venetoclax might be an option for patients already receiving HMA therapy expanding the plethora of options for R/R patients. 

Various mechanisms of resistance to venetoclax have been identified so far and mainly involve MCL-1 or BCL-X_L_ upregulation (Figure 1). Thus, targeting MCL-1 represents a way to overcome the resistance of venetoclax refractory AML either through direct or indirect inhibitors (e.g., targeting MDM2, E3 ubiquitin-protein ligase) [72]. In particular, tp53 apoptotic machinery has been identified as the key to counteract resistance to BCL-2 inhibition. Using a CRISPR/Cas9 system, Nechiporuk et al. showed that knockout of TP53, BAX, and PMAIP1 (Phorbol-12-Myristate-13-Acetate-Induced Protein 1) genes resulted in BCL-2 inhibitors refractoriness in AML cell lines. Moreover, the same study also showed a metabolic change in resistant AML clones which was indicative of a high proliferation rate and energy production deriving from nucleotides synthesis, as well as fatty acids and proteins used for membrane assemblage [73]. Finally, new BCL-2 inhibitors are being developed and clinical trials evaluating their safety and efficacy are ongoing [60].

## 5. Hedgehog Pathway Inhibitors

Hedgehog (HH) signaling pathway is fundamental in embryonic development and homeostasis of adult tissues [74]. HH intertwines with multiple ligands and receptors and is responsible for the segment polarity of developing tissues. In particular, the interaction with protein patched homolog 1 receptor (PTCH1) releases Smoothened (SMO) leading to upregulation of glioma-associated oncogene (GLI) proteins. These proteins finally increase the transcription of HH target genes, involved in cell cycle and proliferation [75]. HH is also important during normal hematopoiesis. Indeed, this process requires intact HH signaling with functions depending on the developmental stage and cell type [76]. The aberrant activation of HH pathway has been first related to malignant transformation in the context of Gorlin–Goltz syndrome, also called nevoid basal cell carcinoma syndrome (NBCCS), an autosomal dominant disorder characterized by basal cell carcinomas due to the mutation of PTCH1 [77]. This aberrant activation has been related also to myeloid malignancies such as MDS and AML where HH signaling seem to be implicated in resistance/disease progression through maintenance of a dormant LSC state [78]. In AML, GLI expression has been associated with *FLT3* mutational status and a negative impact on EFS, relapse-free survival, and OS (*p* = 0.037, 0.026, and 0.013, respectively) [79]. Thus, HH signaling has represented a new target for AML treatment. Fukushina et al. showed that PF-913, an HH pathway inhibitor, elicited a more pronounced effect on the staminal cell compartment rather than on proliferating leukemia cells. Moreover, this compound sensitizes AML blasts to cytarabine arabinoside treatment, overcoming chemotherapy refractoriness [78]. This agent is now known as glasdegib and has been approved in 2018 in combination with LDAC for the treatment of newly diagnosed AML in older adults (age >75) or in patients with contraindications to chemotherapy [80]. The first study using glasdegib provided safety and pharmacokinetic data and recommended a dose of 200 mg or lower once daily, showing activity in many myeloid disorders including MDS and AML [81]. The promising therapeutic potential of this drug was first studied in a combination trial of glasdegib 100 or 200 mg once daily continuously for 28 days together with LDAC (arm 1), decitabine (arm 2), or induction chemotherapy (arm 3) [82]. The results provided data for the recommended phase II dose of 100 mg for glasdegib. This led to the BRIGHT AML 1003 study, an open-label, multicenter trial in which patients older than 75 with AML and higher-risk MDS, not suitable for standard chemotherapy or aged >55 with unfitness criteria, were given glasdegib 100 mg oral once daily (28 days continuously) plus LDAC (20 mg subcutaneously BID) or LDAC alone [83]. OS for the combination arm was 8.3 months with a CR rate of 18.2% whereas for LDAC data showed 4.3 months and 2.6%, respectively. This study led to FDA approval for AML but not for MDS because of the small number of patients enrolled with this latter condition [80]. The expansion phase 2 trial of glasdegib plus LDAC showed similar results. Multiple trials are ongoing to evaluate this compound in combination with induction chemotherapy or other agents. In particular, the BRIGHT AML 1019 (NCT03416179) is a phase III, randomized (1:1), double-blind, placebo-controlled global trial evaluating oral glasdegib 100 mg quaque die plus standard chemotherapy (3+7) or azacitidine in adults with untreated AML [84]. The most common adverse effects ranged from gastrointestinal (transient or resolved with appropriate therapy) to pulmonary infections, QTc prolongation, and hematological toxicity [83]. Glasdegib is metabolized by CYP3A4 and concerns have been formulated for drug interactions with antifungal agents. Finally, as expected if considered its segment polarity function, FDA highlighted its potential risk for embryofetal development with a warning box for this particular toxicity [80]. 

As aforementioned, GLI expression has been related to AML survival outcomes and therefore may be a useful biomarker of HH pathway inhibitors response/resistance. Acquired mutations in SMO gene may cause resistance as suggested by many observations derived by the clinical experience of basal cell carcinoma (BCC) patients. In particular, some case reports showed that arsenic trioxide (ATO), well-known for APL treatment, may overcome HH inhibitors resistance in combination with itraconazole in patients with BCC [85]. Finally, other HH signaling inhibitors such as vismodegib, sonidegib (NCT01826214), taladegib, and itraconazole (also well-known for its antifungal activity) are under experimentation in hematological malignancies.

## 6. Epitherapy 

Epigenetic and genetic anomalies cooperate in AML initiation and progression, as demonstrated by the presence of frequent mutations in genes encoding proteins that control the epigenome and/ or by the abnormal expression of epigenetic proteins. Given the reversible nature of epigenetic changes, targeting epigenetic alterations by using epigenome-influencing agents has represented a powerful approach in the treatment of AML with the promise of restoring the function of affected genes and reprogramming the state of the cells from abnormal to normal [86,87]. This notion relies on the flexibility of the genome. While in normal hematopoiesis, the epigenome easily changes from an embryonic to a differentiated cellular state, in AML and in cancer in general the epigenome can block cells to differentiate constantly. 

In the late 1970s, reverse epigenetic agents amenable to reverse DNA methylation were discovered. However, these agents only reached clinical trials about twenty years later specifically in MDS. The therapeutic strength of epigenetic therapies was empowered by the fact that these agents improved patient tolerance at much low doses. DNA methyltransferase inhibitors were indeed internationally approved by the FDA for the treatment of patients with high-risk MDS and AML less than 30% blasts. More recently these drugs were also approved by EMA (European Medicines Agency) for AML with more than 30% of blasts and in combination with venetoclax for the management of elderly AML ineligible for high-intensity chemotherapy [88,89]. 

The DNA methylation process depends on the function of five enzymes called DNA methyltransferases (DNMTs: DNMT1, DNMT2, DNMT3A, DNMT3B, DNMT3L) which are known to add an extra methyl group to DNA sequences. These enzymes are key players in AML pathogenesis. In particular alterations of *DNMT3A* through somatic mutations are frequent in AML and independently associated with a poor outcome. Recently, *DNMT3A* mutations have been identified also in individuals carrying clonal hematopoiesis of indeterminate potential, a condition associated with risk of developing blood cancers including AML. *DNMT1* is involved in cellular differentiation by methylating hemi-methylated sites after DNA replication and leading to hypo or hypermethylation. In fact, DNMT1 is a target of treatment in AML [90,91]. Nucleoside analogs so called DNA-hypomethylating agents 5-azacitidine (5-AZA or Vidaza*®*) and 5-aza-2′-deoxycytidine (DAC or Dacogen*®*) irreversibly inhibit the enzymatic functions of DNMTs and cause their proteasomal degradation by freely incorporating into DNA (DAC) and RNA (5-AZA) and depleting DNMTs. 

**Figure 2:**
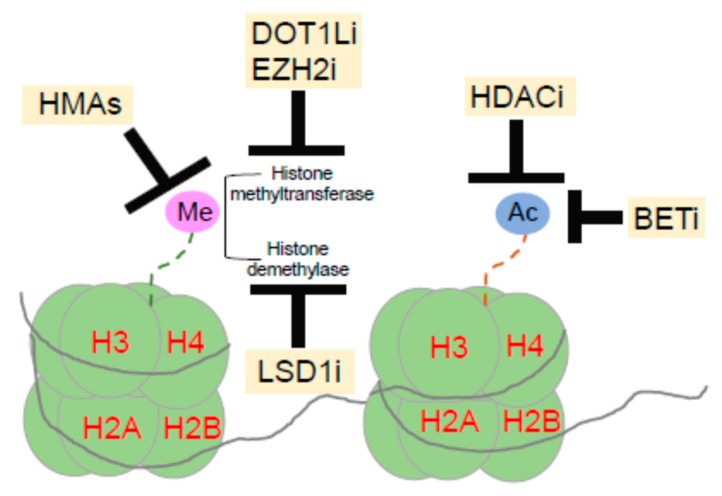
Epigenetic drugs influence main post-translational modifications of the nucleosomal histones including deacetylation of lysine residues (HDACi), methylation (HMAs), reduction of H3K79 methylation (DOT1Li) and H3K27 (EZH2i), demethylation of lysine residues (LSD1i) and bromodomain, and extra-terminal motif inhibitor (BETi). Abbreviations: Me: Methylationl Ac: Acetylation; HDAC: Histone deacetylase; HMA: Hypomethylating agents; DOT1L: Disruptor of telomeric silencing 1-like; EZH2: Enhancer of Zeste 2 Polycomb Repressive Complex 2 Subunit; LSD1: Lysine specific demethylase 1.

The second class of epigenetic drugs are histone deacetylase (HDACs), a group of enzymes capable of removing acetyl groups on lysine residues in the N-terminal tail and on the nucleosome-core of histones and representing the ‘’epigenetic erasers’’ together with TET family of proteins. Deacetylated histones condense the chromatin structure reducing gene transcription levels. Additional drugs targeting epigenetic modifiers are the inhibitors of histone methyltransferases (HTMs) EZH2 and DOT1L, the histone demethylases LSD1, and proteins interacting with acetylated histones (BETs) (see also Figure 2). 

Given the exciting results in cellular models of AML, all these drugs have started early-phase clinical trials [92]. Although HMAs are the standard of treatment only approximately half of patients respond to the treatment with a variability in the duration of response. Alternative approaches for patients failing HMAs are not the standard of care. Several agents are under clinical experimentation [93]. The next-generation DNA hypomethylating agent guadecitabine (SGI-110) was originally produced to resist degradation inferred by cytidine deaminase and extend the exposure of tumor cells to DAC. This drug has been shown to promote tumor associated antigens and sensitize tumor cells to immunotherapy. A single arm phase II study of guadecitabine (SGI-110) in previously untreated patients with intermediate-2 or high-risk MDS showed a median OS of 15 months and median EFS of 14 months. The ORR was 61% (53 patients) including a 22% CR (19 patients) and 3% CR with incomplete platelet recovery (three patients) [94]. Rigosertib (ON 01910.Na) developed by Onconova Therapeutics is a class of sulfones blocking RAS signaling by serving as a RAS mimetic which binds to the RAS-binding domain. A phase 1/2, multicenter, dose-escalating study in patients with MDS and AML is evaluating the combination of oral rigosertib and 5-AZA. The ORR was 68% for 31 patients evaluable for response, including 79% for 14 patients who had never been exposed to HMAs, and 59% for 17 patients who were relapsed or refractory to prior HMAs treatment (NCT01926587). Pevonedistat (MLN4924), a neddylation inhibitor is being evaluated in a phase 3 randomized trial in combination with 5-AZA in patients with higher-risk MDS, CMML, and AML [95]. Epigenetic drugs have effects on several cellular compartments including cytoplasm and nucleus and in many diverse pathways (e.g., cell cycle, apoptosis, immune response). If on one side the pleiotropic effects of epigenetic drugs represent a therapeutic limitation, on the other hand this characteristic has represented the basis for combination therapies. This has been the case of 5-AZA nucleosides restoring the expression of cyclin-dependent kinases inhibitors (e.g., CDKN2A/p16^INK4^), inducing apoptosis by upregulating TRAIL receptor-1 (TNF-related *apoptosis*-inducing ligand), repressing co-stimulatory molecules and molecules functioning in immunological recognition (e.g., HLA class I antigens) and blocking T-cell response through increasing the expression of ligand for checkpoint inhibitor receptors [96].

In fact, combination strategies with other epigenetic drugs have been tested in the clinic; for instance, HDAC inhibitors in combination with HMAs. However, to date randomized studies have showed challenges including an antagonist effect possibly due to lack of clinical benefits. Given also the complexity of the epigenome, drug pharmacodynamics and schedule of administration might represent the biggest barriers to overcome in order to get the maximum efficacy of the agents. Examples of these combinations are exemplified by 5-AZA combined with HDAC inhibitors (entinostat or vorinostat) which showed similar results without leading to any benefits compared to single-agent 5-AZA. This might be due to increased toxicity attributable to combination therapy or to the fact that HDAC inhibitors have a wide number of biological targets which can generate diverse effects. Additional trials are ongoing with the next generation of HDAC inhibitors belinostat, panobinostat, and pracinostat [92,97]. The latter has been combined with 5-AZA in a phase 2 open-label, single-arm, two-stage, multicenter trial of patients with newly diagnosed AML (age 65 years or older) (NCT01912274). Treatment schedule was: 60 mg of pracinostat, three times a week, three weeks followed by one week of break repeated every 28 days; 75 mg/m^2^ 5-AZA for the first seven days of each 28-day cycle, SC or IV according to individual tolerability. This study showed an overall composite CR of 52% (which is higher than 5-AZA alone). A phase 3 trial with both agents is now ongoing (NCT03151408). Combination of HMAs with the LSD1 inhibitor NCB059872 (NCT02712905) or with the PRTM5 inhibitor GSK3326595 (NCT03614728) is now under evaluation in clinical trials. HMAs have also been combined with immunotherapy agents as the case of DAC plus avelumab targeting DNMT and PD-L1 (NCT03395873) or 5-AZA plus nivolumab targeting DNMT and PD-1 (NCT0385367).

Histone acetyltransferases (HATs) small molecule inhibitors are also being tested. HAT are enzymes capable of transferring an acetyl group from acetyl coenzyme A to the ε-amino group of lysine residues in histones. Type A HATs consist of three families of enzymes (p300/CBP, GNATs, a MYST) while type B HATs consist of KAT1. HAT inhibitors include bisubstrate inhibitors, natural compounds, and low molecular weight inhibitors. This is the case of the HAT paralogs p300 and CREB binding protein (CBP) inhibitors. Both p300 and CBP have oncogenic roles in AML and other malignancies. CCS1477 is a small molecule which binds selectively to p300/ CBP, decreases cellular proliferation of blast cells and upregulates differentiation-promoting markers (e.g., CD11, CD86). In AML cell lines derived xenografts, CCS1477 (20 mg/Kg) reduces tumor burden [98]. CCS1477 was tested in an open-label phase I/IIa trials in patients with advanced hematologic malignancies. Study design and eligibility criteria were presented at the past American Society Hematology annual meeting [99]. 

Inhibitors of the lysine acetyltransferases (KATs) have also been studied. Among several KATSs *KAT6A* plays key roles in normal hematopoiesis and is target of chromosomal translocations in AML.

Studies have shown that KAT6A blocks senescence by regulating *CDKN2A*. Similarly, KAT6B has also being selected for targeted approaches as indicated by the development of potent KAT inhibitors (WM-8014 for KAT6A) and WM-1119 (KAT6B) [100].

## 7. Tp53 Pathway Inhibitors

Tp53 signaling pathway is essential for cell cycle regulation, senescence, and apoptosis [101]. About 5–15% of patients with AML harbor mutations in the TP53 tumor suppressor gene [102]. However this incidence rises to 60–70% when considering the subset of patients with complex cytogenetic profile, which are also characterized by particular dismal outcomes. [46,103] PRIMA-1(APR-017) and its methylated derivative PRIMA-1^MET^, also called APR-246, specifically target mutant tp53, reactivating its function and showing an antileukemic activity in vitro models [104]. Chemically, APR-246 modifies the core domain of mutated tp53 through alkylation of thiol groups. Particularly, APR-246 is effective in AML cell lines, as well as in primary AML patient cells alone and in combination studies with chemotherapeutic drugs where it shows synergistic activity with daunorubicin and azacitidine, among others [105]. A first-in-human study demonstrated safety and tolerability of APR-246 in hematological malignancies and prostate cancer, determining its maximum-tolerated dose (MTD) as 60 mg/kg and pharmacokinetics [106]. In another study of the same group, APR-246 has been used in a cohort of patients with AML and CLL with deletion of 17p not eligible for other therapies with a new schedule of treatment and dose regimen of 67.5 mg/kg, given as 6 h infusion on four consecutive days, instead of a 2 h infusion of the previous first-in-human study [107]. Based on these promising results, this compound has been tested in ongoing clinical trials in combination with other agents such as azacitidine and venetoclax (NCT03745716, NCT04214860, NCT03588078). Additional in vitro studies supported clinical trials with small-molecule MDM2 inhibitors in AML. Examples of these compounds are AMG-232, RG-7388/RO-5503781/Idasanutlin and DS-3032b/Milademetan.

## 8. Conclusions and Future Perspectives

Only about 30% of AML patients become long-term survivors. Still there is a paucity of pharmacologic agents for patients who relapsed or become refractory to treatment. Especially in case of unfit-for-chemotherapy patients, a new paradigm is impelling: A comprehensive targeted molecular study is needed to better choose the best tailored therapy to avoid unnecessary toxicity. Thus, not only younger patients but also elderly patients may be suitable of NGS-based approaches and benefit of a deeper molecular characterization of their disease. The availability of these new orally antileukemic compounds is rendering feasible treating also this setting of frail patients where the aim is now also a hematological improvement with transfusion independence and not only the achievement of a CR. Moreover, new insights are giving the rationale for studies on maintenance therapy in AML and this field may be of paramount interest in elderly patients, characterized by more aggressive AML phenotype and possibly taking advantage from a less intense and more prolonged treatment schedule. A lot of efforts have been made to test new drugs and their combination with existing agents (see also Table 2). The results of several clinical trials will summarize the benefits and efficacy of those combinations. Translational research based on better cellular and pre-clinical models and the understanding of the mechanisms of action of novel agents will altogether serve to overcome challenges and guide clinical decisions. 

## Figures and Tables

**Table 1:** Adapted from Short et al. [19] FLT3 inhibitors characteristics. Abbreviations: AXL: Tyrosine-protein kinase receptor; bid: Two times daily; c-KIT: Proto-oncogene receptor tyrosine kinase; GI: Gastrointestinal; PDGFR: Platelet-derived growth factor receptor; PKC: Protein kinase C; RAF: Rapidly Accelerated Fibrosarcoma serine/threonine-protein kinase family kinase; RET: RET proto-oncogene tyrosine kinase; tid: Three times daily; TKD: Tyrosine kinase domain; VEGFR: Vascular endothelial growth factor receptor.

FLT3 Inhibitor	Type	Non FLT3 Targets	TKD Activity	Dose	Major Toxicities
Sorafenib	II	cKIT/PDGFR/RAF/VEGFR	No	400 bid	myelosuppression, rash, hemorrhage
Midostaurin	I	cKIT/PDGFR/PKC/VEGFR	Yes	50 bid	GI toxicity, myelosuppression
Quizartinib	II	cKIT/PDGFR/RET	No	30–60 mg die	QTc prolongation, myelosuppression
Crenolanib	I	PDGFR	Yes	100 mg tid	GI toxicity
Gilteritinib	I	AXL	Yes	120 mg die	Diarrhea, transaminases increase

**Table 2:** FDA approval status for AML of major antileukemic agents discussed in the present manuscript.

Drug	Targeted Signaling Pathways ^1^	Status ^2^
FLT3 inhibitors		
Sorafenib	FLT3, c-KIT, PDGFR, RAF, VEGRF	Approved
Midostaurin	FLT3, c-KIT, PDGFR, PKC, VEGFR	Approved
Quizartinib	FLT3, c-KIT, PDGFR, RET	Investigational
Crenolanib	FLT3, PDGFR	Investigational
Gilteritinib	FLT3, AXL	Approved
IDH inhibitors		
Ivosidenib	IDH type 1	Approved
Enasidenib	IDH type 2	Approved
Olutasidenib	IDH type 1	Investigational
Vorasidenib	IDH type 1 and 2	Investigational
BCL-2 pathway inhibitors		
Venetoclax	BCL-2	Approved
Hedgehog pathway inhibitors		
Glasdegib	Inhibition of Smoothened (SMO)	Approved
Vismodegib	Inhibition of Smoothened (SMO)	Investigational ^3^
Sonidegib	Inhibition of Smoothened (SMO)	Investigational ^3^
Taladegib	Inhibition of Smoothened (SMO)	Investigational
Epigenetic drugs		
Azacitidine	Inhibition of DNA methyltransferase	Approved
Decitabine	Inhibition of DNA methyltransferase	Approved
Guadecitabine	Inhibition of DNA methyltransferase	Investigational
Rigosertib	Ras/Raf/MAPK Pathway inhibitor	Investigational
Pevonedistat	Selective NEDD8 inhibition	Investigational
Pracinostat	HDAC Inhibitor	Investigational
Panobinostat	HDAC Inhibitor	Investigational ^4^
Belinostat	HDAC Inhibitor	Investigational ^5^
Tp53 pathway inhibitors		
APR-246	Tp53	Investigational
Idasanutlin	Mdm2	Investigational
Milademetan	Mdm2	Investigational
Others		
Daunorubicin	Topoisomerase II	Approved
Cytarabine	Inhibition of DNA polymerase	Approved

AXL: Tyrosine-protein kinase receptor; BCL-2: B-cell lymphoma 2; c-KIT: Proto-oncogene receptor tyrosine kinase; HDAC: Histone deacetylases; IDH: Isocitrate dehydrogenase; Mdm2: E3 ubiquitin-protein ligase; NEDD8: Neural precursor cell expressed developmentally downregulated 8; PDGFR: Platelet-derived growth factor receptor; RAF: Rapidly Accelerated Fibrosarcoma serine/threonine-protein kinase family kinase; Tp53: Tumor Protein 53; VEGFR: Vascular endothelial growth factor receptor. ^1^ Gene symbol and official names follow the nomenclature of the GeneCards: The Human Gene Database; ^2^ According to FDA https://www.fda.gov/; ^3^ Approved for Basal Cell Carcinoma; ^4^ Approved for Multiple Myeloma; ^5^ Approved for peripheral T-cell lymphoma.
